# A digital polymerase chain reaction method targeting non-recombined T-cell receptor sequences aids in diagnosing primary cutaneous T-cell lymphomas

**DOI:** 10.7150/jca.136694

**Published:** 2026-07-13

**Authors:** Chien-Chin Chen, Cheng-Lin Wu, Bo-Jiun Tzeng, Pin-Jun Chen, Wan-Li Chen, Sern Yan Lim, Yi-Ling Chen, Yi-Lin Chen, Chung-Liang Ho, Tsunglin Liu

**Affiliations:** 1 Department of Biotechnology and Bioindustry Sciences, College of Bioscience and Biotechnology, National Cheng Kung University, Tainan, Taiwan.; 2 Department of Pathology, Ditmanson Medical Foundation Chia-Yi Christian Hospital, Chiayi, Taiwan.; 3 Department of Cosmetic Science, Chia Nan University of Pharmacy and Science, Tainan, Taiwan.; 4 Doctoral Program in Translational Medicine, National Chung Hsing University, Taichung, Taiwan.; 5 Department of Pathology, National Cheng Kung University Hospital, College of Medicine, National Cheng Kung University, Tainan, Taiwan.; 6 Institute of Molecular Medicine, College of Medicine, National Cheng Kung University, Tainan, Taiwan.; 7 Molecular Medicine Core Laboratory, Research Center of Clinical Medicine, National Cheng Kung University Hospital, Tainan, Taiwan.; 8 Medical Laboratory Science and Biotechnology, College of Medicine, National Cheng Kung University, Tainan, Taiwan.

**Keywords:** BIOMED-2, cutaneous T-cell lymphoma, digital PCR, T-cell receptor

## Abstract

**Background:**

Diagnosing primary cutaneous T-cell lymphoma (PCTCL) relies on the comprehensive evaluation of clinical presentation, histopathology, immune profile, and molecular testing. Regarding molecular testing, the BIOMED-2 assay is most commonly used for detecting monoclonality of T-cell receptor (TCR) genes. However, its sensitivity is not ideal for PCTCLs, and a supplementary test is required. We recently found that the ratios of non-recombined TCR sequences J2-2P and J2-3 of the *TCRβ* gene were higher in T-cell lymphoma (TCL) samples than in non-TCL cases. Here, we aim to evaluate the diagnostic value of this novel biomarker for PCTCL via digital polymerase chain reaction (dPCR) as a supplementary method to the BIOMED-2 clonality assay.

**Methods:**

Formalin-fixed paraffin-embedded tissue samples of clinicopathologically proven PCTCL cases with a negative BIOMED-2 clonality result were retrospectively retrieved, and the clinicopathological characteristics were collected. After RNA extraction and quality checking, dPCR was performed on PCTCL specimens and control samples to quantify the J2-2P and J2-3 sequences.

**Results:**

We collected 35 skin biopsy samples from patients with early-stage PCTCL and 11 inflammatory dermatoses as controls. Ratios of the J2-2P to J2-3 concentration in controls ranged from 0.03 to 0.55% (mean ± standard deviation: 0.21 ± 0.17%). In contrast, 25 of the 35 (71.42%) PCTCL specimens exhibited J2-2P/J2-3 ratios exceeding the defined upper limit of the control group.

**Conclusion:**

Our dPCR assay on the non-recombined *TCRβ* sequences revealed that a majority of the PCTCL cases with a negative BIOMED-2 result had higher ratios of the J2-2P to J2-3 concentration than inflammatory dermatoses. Therefore, this approach may supplement the BIOMED-2 clonality assay for diagnosing PCTCL.

## 1. Introduction

Primary cutaneous T-cell lymphoma (PCTCL) is characterized by neoplastic T cells that develop primarily in the skin without evidence of systemic or extra-cutaneous involvement 1-3. In the US National Cancer Registry data, 14,942 new PCTCL cases were diagnosed between 2000 and 2018, with an incidence of 8.55 per million 4. PCTCLs are a heterogeneous set of lymphoma entities with varied clinical features (such as papules, patches, plaques, nodules, and erythroderma) and histological features (including intraepidermal, dermal, or subcutaneous lymphocyte infiltrates with varying cellular sizes and shapes) 1-3,5. Additionally, their immunophenotypic and genetic characteristics are distinct 1,2. The World Health Organization (WHO) has classified PCTCLs into eleven types, including mycosis fungoides (MF), Sézary syndrome, primary cutaneous CD30-positive lymphoproliferative disorders, primary cutaneous gamma-delta T-cell lymphoma, primary cutaneous CD4+ small/medium T-cell lymphoproliferative disorder**,** primary cutaneous acral CD8+ T-cell lymphoma, cutaneous CD8+ aggressive epidermotropic T-cell lymphoma**,** adult T-cell lymphoma/leukemia, systemic chronic active EBV disease, subcutaneous panniculitis-like T-cell lymphoma (SPTCL), and primary cutaneous peripheral T-cell lymphoma, NOS (PTCL) 3. Among all types of PCTCLs, MF is indolent with globally the highest incidence rate (5.42 per million) but can transform into an aggressive leukemia known as Sézary syndrome, with an annual percentage increase of 3.83% 4. Primary cutaneous CD30-positive lymphoproliferative disorders, primary cutaneous CD4+ small/medium T-cell lymphoproliferative disorder, and SPTCL are indolent, with the second to fourth incidences among PCTCLs 3. In Taiwan, MF (56.8%), primary cutaneous CD30-positive lymphoproliferative disorders (14.8%), and SPTCL (3.7%) were the most common PCTCL subtypes, and most PCTCLs were presented at early stages (85.8%) 6.

PCTCL is diagnosed by integrating clinical manifestations, histological characteristics, immunophenotypic patterns, and molecular data. Early diagnosis of PCTCL can be challenging due to its modest clinical presentation, low percentage of tumor cells, small or limited tissue biopsy size, and nonspecific histopathological characteristics. In particular, PCTCLs and reactive lesions can be misdiagnosed because of their similar clinical and histopathological features. In these cases, molecular tests aid in diagnosing PCTCL 5, highlighting the importance of integrating various evidence 7-8. The International Society for Cutaneous Lymphomas (ISCL) has suggested an algorithm to diagnose early-stage MF. The diagnosis involves a careful evaluation of histopathological features, clinical symptoms, immunohistochemical tests, and molecular tests, including examination of T-cell receptor (TCR) gene rearrangements 9-11.

For the molecular diagnosis of PCTCL, the BIOMED-2 multiplex polymerase chain reaction (PCR) assay is currently the most widely used molecular test worldwide. It examines the monoclonality of TCR rearrangement, whose existence supports the diagnosis of malignant T-cell lymphoma (TCL) 12-13. However, for diagnosing PCTCL, the BIOMED-2 assay has variable sensitivities 14, e.g., 66% in a Netherlands study 15, 76% in Taiwan 16, and 78% in a German cohort 17. Therefore, a supplementary test using a novel biomarker may help enhance the molecular test for PCTCL.

**Figure 1:**
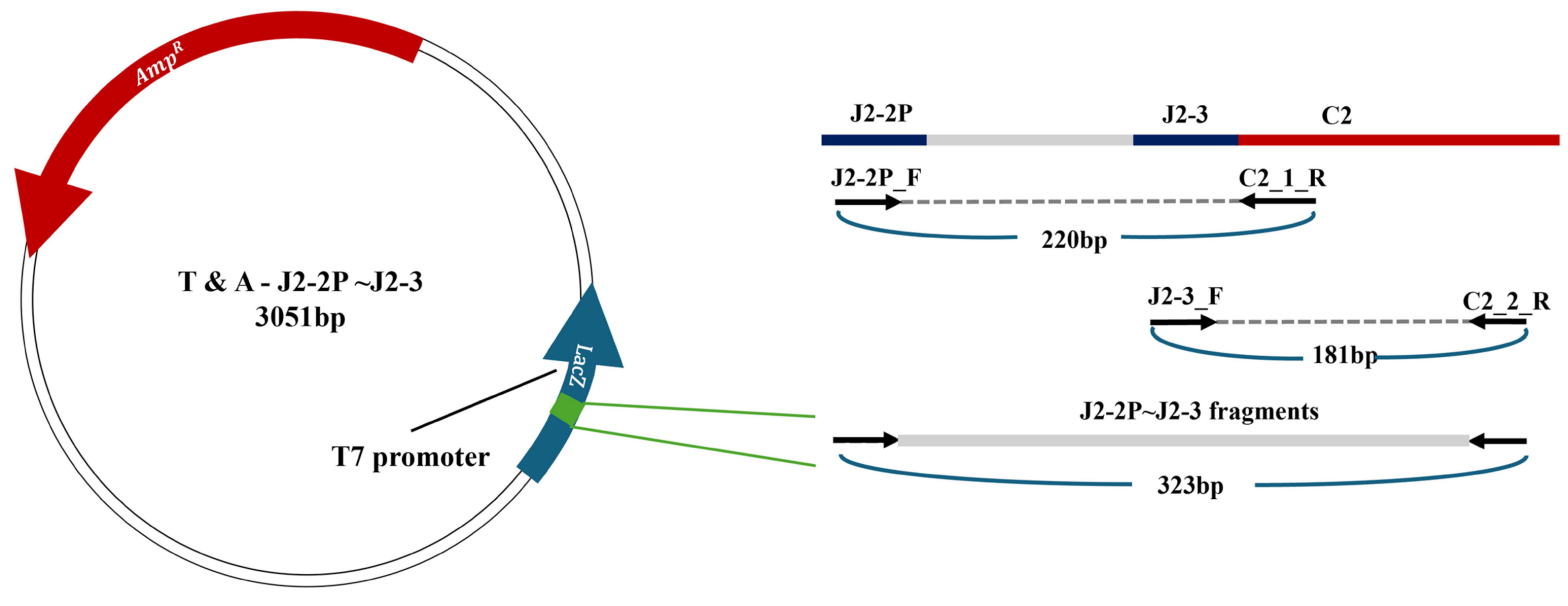
The J2-2P~J2-3:C2 fragment of the plasmid construct. To construct the fragment, two sets of primers, J2-2P_F/C2_1R and J2-3_F/C2_2R, are used to amplify the mRNA containing the J2-2P and J2-3 gene segments separated by an intergenic region and concatenated to the C2 gene segment (top object on the right). In addition to the two amplicons of 220 bps and 181 bps respectively, the PCR results in longer amplicons starting from J2-2PF to C2_2R (323 bp). The longest amplicons are isolated via gel electrophoresis and inserted to the T&A clone.

We recently found the prevalence of non-recombined *TCRβ* sequences in healthy individuals and TCL patients 18. A non-recombined TCR sequence contains a J gene segment but not a D and V gene segment. Interestingly, the ratios of the non-combined TCRβ sequences, specifically J2-2P and J2-3 sequences, were higher in the TCL patients than in the non-TCL cases, suggesting a novel biomarker for supplementing the diagnosis of TCL. J2-2P is a pseudogene segment in front of the J2-3 gene segment and is used here to denote a stretch from the J2-2P to the J2-3 gene segment, i.e., J2-2P~J2-3 (Figure 1), which is always non-recombined. A J2-3 sequence is defined as a *TCRβ* sequence that contains the J2-3 gene segment, which can be present in either a J2-2P sequence or a recombined *TCRβ* gene involving J2-3, such as V1:J2-3. Therefore, J2-2P is included in J2-3, and the ratio of the J2-2P to J2-3 concentration should be less than one. Here, we examined whether this novel biomarker could also supplement the diagnosis of PCTCL. In particular, we investigated PCTCL cases with both clinical and histopathological evidence and demonstrated a negative BIOMED-2 result, indicating potential false negatives. We applied digital PCR (dPCR) to quantify the J2-2P to J2-3 concentration ratio and compared the results with those of control samples. The current study found that approximately 70% of the false-negative PCTCLs in the BIOMED-2 assay had a higher J2-2P/J2-3 ratio in dPCR, indicating its potential utility as a supplementary method.

## 2. Materials and Methods

### 2.1 Plasmid construction and determining the limit of detection (LoD) in dPCR

For accurate quantification, we constructed a plasmid containing the J2-2P~J2-3 gene segment of the *TCRβ* gene concatenated to the C2 gene segment (Figure 1). This construct served as a positive control in subsequent dPCR assays. The J2-2P~J2-3:C2 and J2-3:C2 segments were amplified via PCR using two distinct primer sets: J2-2P_F: 5'-AGGCGCTGCTGGGCGTCT-3' / C2_R: 5'-GGGTGGGAACACGTTTTTCAGG-3' for the J2-2P~J2-3:C2 segment and J2-3_F: 5'-GCACAGATACGCAGTATTTTGG-3' / Cβ2_R: 5'-TCAGCTCCACGTGGTCGGGGT-3' for the J2-3:C2 segment, respectively. Each PCR reaction was performed in a 25 μL volume under the following thermal cycling conditions: an initial denaturation at 95 °C for 5 minutes, followed by 35 cycles of 95 °C for 30 seconds, 60 °C for 60 seconds, and 72 °C for 60 seconds. A final extension step at 72 °C for 10 minutes completed the amplification, after which the reaction was held at 12°C. The PCR resulted in three amplicon products and the largest one (323 base pairs) was isolated via gel electrophoresis. The largest product was subsequently cloned into a T&A Cloning Vector (Geneaid, Taipei, Taiwan). The integrity of the cloned product was confirmed through Sanger sequencing. The copy number was calculated based on the concentration of plasmid DNA and its fragment size. This validated fragment was subsequently used as a positive control in our dPCR experiments.

To determine LoD for the J2-2P and J2-3 sequences, the plasmids were serially diluted 10-fold, generating standards ranging from 10^6^ to 10^0^ copies. The 10^0^-10^6^ copies of the plasmid were quantified by dPCR to evaluate the concentrations using the two primer sets, and the data were plotted to build a standard curve.

### 2.2 Collection of PCTCL specimens from clinical patients

This clinical study was approved by the Institutional Review Board of the National Cheng Kung University Hospital (NCKUH) (Number: B-ER-110-042, period of the project: 2021.04.27 to 2025.12.31). The requirement for patient-informed consent was waived because only de-identified cases were used.

We aimed to recruit patients with PCTCLs and negative BIOMED-2 results retrospectively. Inclusion criteria for this study comprised: 1) pathological confirmation of PCTCL, 2) negative TCR clonality as determined by the BIOMED-2 assay, 3) accessible formalin-fixed paraffin-embedded (FFPE) tumor tissue, and 4) availability of traceable and corroborating clinical data. Exclusion criteria for this study encompassed: 1) suboptimal RNA quality, 2) insufficient specimen quantity, 3) a positive TCR clonality from the BIOMED-2 assay, or 4) clinicopathological findings not fulfilling the diagnostic criteria for PCTCL.

From January 2019 to December 2024, 147 FFPE tissue samples of clinically suspicious PCTCL were requested for a BIOMED-2 test of TCR monoclonality at NCKUH. Forty-nine samples with a positive BIOMED-2 test (monoclonality shown in either TCR gamma or beta chain) were assumed to be consistent with PCTCLs and excluded because a supplementary test was unnecessary. Among the 98 samples with a negative BIOMED-2 test, we further excluded 3 recurrent samples to avoid repetitive calculations for the same patients, 25 samples with poor RNA quality, and 35 samples without histological evidence of PCTCL, as determined by reviewing the histopathology, immunophenotypes, and clinical outcomes. These specimens fulfilled the diagnostic criteria of the fifth edition of the WHO classification of lymphoid neoplasms for PCTCLs 2,3 but showed a polyclonal or equivocal result for the *TCRγ* and *TCRβ* genes in the BIOMED-2 assay. As a result, we identified 35 specimens from 35 patients as our experimental group.

These 35 specimens comprised 27 MF, 4 PTCL, and 4 SPTCL cases. Diagnosis, tumor proportion, and disease status (naïve, recurrent, or post-treatment) were further confirmed through histopathology and chart review. MF was diagnosed using the ISCL algorithm, and a total of 4 points from clinical, histopathological, molecular, and immunopathological factors were required 9. PTCL and SPTCL were diagnosed according to the updated WHO classification criteria 2. The detailed information is listed in Table 1. In addition, 11 FFPE tissues from skin samples of 11 patients with inflammatory dermatoses were retrieved from the pathology tissue bank at NCKUH as control samples.

### 2.3 RNA extraction and RNA quality control

For PCTCL cases with a tumor percentage equal to or greater than 20%, we would cut five whole FFPE tissue slices per case, each with a thickness of 5 μm. For the remaining PCTCL cases with a tumor percentage of less than 20%, we would cut five blank tissue slides of 5 μm thickness, circle the area with a higher tumor percentage, and perform macro-dissection under the pathologist's observation. Then, the macrodissected tissues were sent for RNA extraction.

Tissues from whole FFPE tissue slices or macro-dissection were placed in a 1.5 mL tube. We used xylene and 100% ETOH to de-wax and dehydrate the specimen, put them in a 37 °C dry bath to evaporate the alcohol, and then used the Qiagen RNeasy FFPE Kit (Qiagen, Hilden, Germany) to extract RNA. In the entire RNeasy FFPE procedure, we added FFPE specimens with 10 μL proteinase K and 240 μL Buffer PKD, mixed them well, heated at 56°C and 80°C for 15 minutes each, ice bathed for 3 minutes, centrifuged at 10 °C, 13,500 rpm for 15 minutes, aspirated the supernatant, and then added DNase Booster Buffer 25 μL and 10 μL DNase reacting at room temperature for 15 minutes to remove genomic DNA effectively. After adding 500 μL Buffer RBC and 1,200 μL 100% ETOH, the solution was put into RNeasy MinElute spin column to purify concentrated RNA, centrifuged at 10 °C, 12,000 rpm for 30 seconds, washed twice with 500 μL Buffer RPE, centrifuged at 10 °C, 12,000 rpm for 30 seconds, and then idled for 3 minutes. Finally, 20-25 μL of RNase-free water was added to the solution, and the concentration was determined using a Nanodrop (Thermo, Massachusetts, USA). For subsequent experiments, the RNA concentration ranged from 7 to 776 ng/µL.

### 2.4 Reverse transcription-PCR (RT-PCR) and complementary DNA (cDNA) synthesis

Synthesis of first-strand cDNA was performed through the following steps. Step1: take 500 ng RNA into a 0.2 mL tube, add H_2_O to make up the volume to 5 μL, and set PCR conditions as shown in Supplementary Table S1; step2: add related reagents for first-strand cDNA syntheses (Supplementary Table S2), react, and PCR conditions are shown in Table S3. To ensure RNA quality, we used GAPDH (165bp) and β2-microglobulin (256bp) as housekeeping genes for internal control following the same methodology as in our previous publication 19. In brief, if the GAPDH and β2-microglobulin can be successfully amplified, the reverse transcription is reliable and successful, as illustrated in Supplementary Figure S1. Otherwise, additional tissue slices would be cut to re-extract RNA and repeat RT-PCR.

Since FFPE samples are cumbersome to prepare and the quality of extracted RNA is relatively poor, the ReliaPrep™ DNA Clean-Up and Concentration System (Promega, Madison, WI) was used to remove excess nucleotides and primers from cDNA through purification. In brief, we added the reagent Membrane Binding Solution and 100% isopropanol provided by the kit in sequence, mixed the sample thoroughly, loaded the sample into ReliaPrep™ Minicolumn seated in a collection tube, centrifuged at 12,000 rpm for 15 seconds, and then added 200 μL Column Wash Solution (CWE), centrifuged for 15 seconds, then added 300 μL Buffer B (BWB) to wash the column twice, centrifuged for 15 seconds, idled for 1 minute, and finally added 25 μL H2O to dissolve.

### 2.5 dPCR for detecting the non-recombined J2-2P and J2-3 sequences

Clarity™ Digital PCR (JN MEDSYS, Henderson, SG) system, a chip-in-a-tube technology, was used to quantitatively detect the J2-2P and J2-3 concentrations. Regarding the detection mechanism, its wafer is divided into 10,000 holes, and the autoloader is used to disperse DNA sequences into each hole. Ideally, each hole may contain 1 or 0 strands of DNA. Furthermore, as a separate PCR space, the copy number is obtained by detecting the fluorescent signal and converting it to achieve an absolute quantitative method 20.

In general, dPCR adds two sets of different primers and probes into the PCR reaction tubes to detect different targets through the probe label with different fluorescence dyes (FAM: fluorescein, HEX: hexachloro-fluorescein). The operating reagents are shown in Supplementary Table S4. In this study, the above two sets of primers for amplifying the J2-2P and J2-3 sequences were used. The corresponding probes were prepared by Integrated DNA Technologies as previously published (J2-2P probe: 5'-HEX-CTCTCCCAG/ZENCACCCAGAACCAGGA/3IABkFQ/-3'; J2-3 probe: 5'-FAM-CTGACAGTG/ZEN CTCGAGGACCTGAAAAACGT/3IABkFQ/-3') 18. After the specimen was thoroughly mixed and briefly centrifuged, the tube strip, platform, and slider of the JN Clarity™ consumables set (JN MEDSYS, Henderson, SG) were assembled. Following visual confirmation that the wafer was undamaged, the platform was inserted into the tube strip and aligned with the upper edge of the wafer. The wafer was then mounted onto the autoloader, and the slider was positioned on the platform. Mechanical partitioning was subsequently performed, evenly distributing 15 μL of the specimen across the wafer. The tube strip was then transferred to the sealing enhancer to securely seal each sample. Finally, 245 μL of sealing fluid was added to the tube prior to amplification. The dPCR cycling conditions are detailed in Supplementary Table S5.

After the dPCR reaction, we used the Clarity Plus dPCR system (JN MEDSYS, Henderson, SG) to detect whether each hole had a fluorescent signal and convert it to DNA concentration (copies/μL) for the J2-2P and J2-3 sequences as illustrated in Supplementary Figure S2. Ratio of the J2-2P to J2-3 concentration is the metric used to determine disease status, as described in our previous study 18. To avoid underestimation, the concentration of a measurement of zero FAM positive signal was set as 0.035, which was half of the concentration when one FAM positive signal was detected. The entire workflow of the experimental work is illustrated in Supplementary Figure S3.

### 2.6 Statistical analysis

GraphPad Prism 8.01 software (GraphPad Software, San Diego, CA, USA) and Microsoft Office Excel 2016 (Microsoft, Redmond, WA, USA) were used for statistical analysis and data visualization. Data were presented as frequencies, percentages, means, standard deviations, and ranges. Mean ± standard deviation (SD) or median with interquartile range (IQR) or 95% confidence interval (CI) of the mean represents continuous variables, while number [percentage (%)] represents discrete variables. The Mann-Whitney U test was used to analyze two unpaired samples that were not normally distributed. The chi-square test or Fisher's exact test was employed to examine the presence of associations or dependencies between categorized variables. Regression analysis was used to explore the linear relationship between plasmid copy number and the concentrations of the J2-2P and J2-3 sequences. When a *p*-value is smaller than 0.05 (two-tailed), differences were considered statistically significant. In addition, we determined that a higher J2-2P to J2-3 ratio should be more than a three-SD increase relative to the control samples' mean.

## 3. Results

### 3.1 LoD of the J2-2P and J2-3 sequences in dPCR

**Figure 2:**
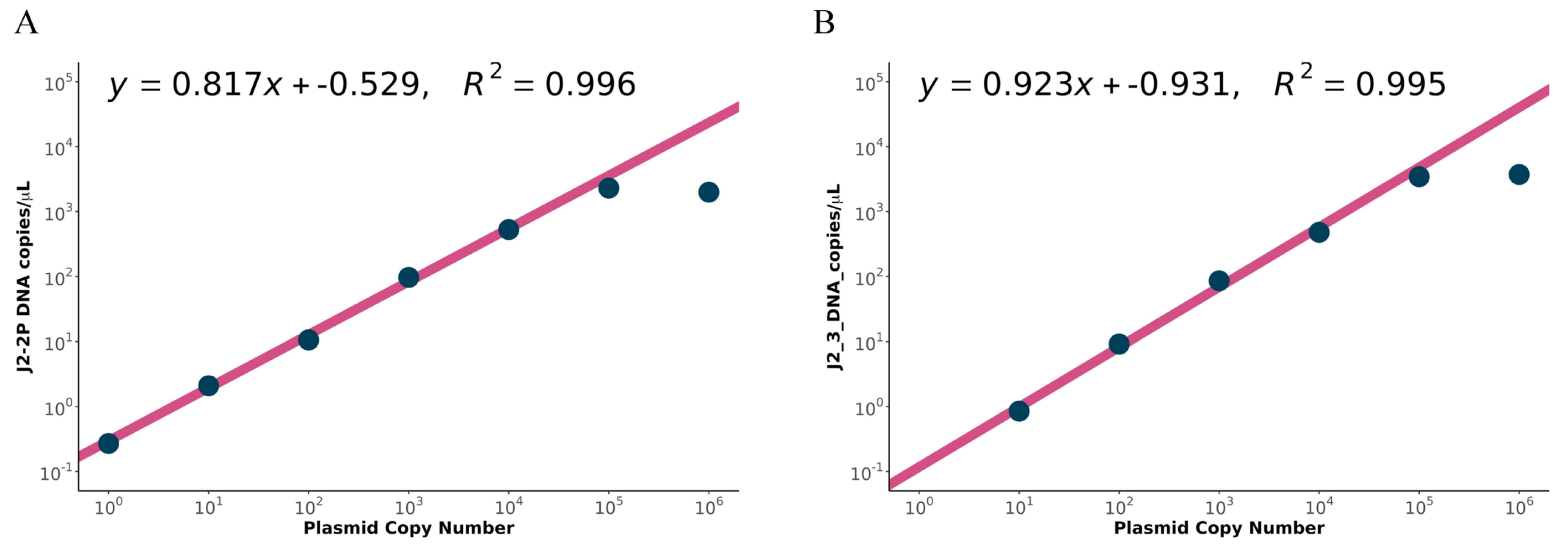
Relationship between plasmid copy number and the concentrations of the (A) J2-2P and (B) J2-3 sequences determined by dPCR. In each plot, the four data points with copy number 10^1^ to 10^4^ are used for linear regression.

Before applying dPCR to clinical samples, we determined the LoD of our targeting sequences. The copy numbers of plasmids containing the J2-2P~J2-3 gene segment were serially diluted from 10^6^ to 10^0^ and quantified by dPCR, and the examination was performed in duplicate. Fluorescent signals of the J2-2P and J2-3 sequences were evenly distributed across the chip and decreased with the dilution (Supplementary Figure S4A). Based on Figure 2, the reliable detection range of plasmid copy numbers for the J2-2P and J2-3 sequences was determined to be 10^1^ to 10^4^ copies. This range was established because fewer than 10^1^ copies yielded no signal in J2-3, while the measurement started to plateau at 10^5^ copies for both the J2-2P and J2-3 sequences (Supplementary Figure S4B). Importantly, these plasmids copy numbers were utilized strictly as a calibration standard to establish the theoretical limit of detection and dynamic range of our primers. While directly comparing plasmid concentrations to FFPE-derived cDNA copies is complex due to matrix effects, the standard curve confirms assay linearity. In terms of the dPCR concentration, a value greater than 10^3^ would suggest saturation and the sample was diluted for a re-measurement.

### 3.2 Patients and tissue characteristics

Based on histopathology, immunophenotype, and clinical outcomes, we collected 35 skin samples from 35 patients confirmed with PCTCL. All patients were clinical stage I and evidence of tumor involvement was restricted to the skin, regardless of their PCTCL types. All the PCTCL specimens showed a negative BIOMED-2 test result, indicating false negatives. The age, gender, histological diagnosis, BIOMED-2 result, biopsy sample size, tumor proportion in histopathology, DNA quality, and RNA concentration were summarized in Table 1. In our cohort, the male-to-female ratio was 1.33:1, with a mean age of 57.91 years. Besides the 4 samples of PTCL and the 4 samples of SPTCL, the remaining 27 samples (77.14%) were of the MF type. The biopsy size ranged from 0.40 to 4.30 cm (mean ± SD: 1.01 ± 0.76 cm), and the tumor proportion was 15.23% on average (SD: 21.06%). The mean extracted RNA concentration was 115.35 ng/µL (SD: 327.70 ng/µL), and all PCTCL samples' cDNA quality was at least 300 bp.

### 3.3 Results of the dPCR assay for J2-2P and J2-3

The PCTCL samples and control tissues were subjected to dPCR to measure concentrations of the J2-2P and J2-3 sequences. To ensure accurate J2-2P/J2-3 ratio calculations, cases exhibiting fewer than five FAM-positive signals of the J2-3 measurement were excluded before further analysis, as the denominator of the ratio was not reliable. Herein, we excluded two PCTCL cases and one control case before further analysis.

In the control group, the J2-2P concentrations varied from 0.035 to 0.07 copies/µL, with a mean of 0.04 copies/µL, while the J2-3 concentrations had a mean of 41.22 copies/µL (range: 0.14-130.76 copies/µL). The ratios of J2-2P to J2-3 averaged 0.21% (range: 0.03-0.55%), and the SD was 0.17%. The dPCR results for the control samples are presented in Table 2. Based on the empirical rule, it is a statistical guideline that describes approximately 99.7% of data points that would fall within three SDs of the mean 21. We determined that 0.72%, which represents the sum of the mean and three standard SDs of the control samples, of the J2-2P/J2-3 ratio represents the upper limit of inflammatory dermatoses.

**Figure 3:**
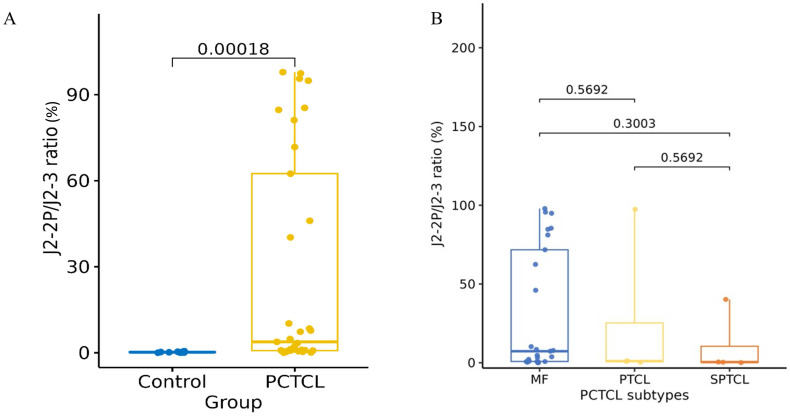
** (A)** The J2-2P/J2-3 ratios between controls and PCTCLs. Using the Mann-Whitney U test, we found that the J2-2P/J2-3 ratios in PCTCL are significantly higher than those in the control group (*p*-value =0.00018, two-tailed). **(B)** The J2-2P/J2-3 ratios stratified by specific PCTCL subtypes including mycosis fungoides (MF), primary cutaneous peripheral T-cell lymphoma, NOS (PTCL), and subcutaneous panniculitis-like T-cell lymphoma (SPTCL), showed no statistically different among three subtypes.

Regarding the PCTCL group, the J2-2P concentrations varied from 0.00 to 664.02 copies/μL (mean±SD: 25.39±111.81 copies/μL; 95% CI: -13.02˗63.80 copies/μL), and the J2-3 concentrations ranged from 0.00 to 681.45 copies/μL (mean±SD: 57.76±129.11 copies/μL; 95% CI: 13.40˗102.1 copies/μL). The ratios of J2-2P to J2-3 had a mean value 27.66% with an SD of 37.97% (range: 0.01˗97.87%; 95% CI: 14.20˗41.13%) (Table 1). Figure 3A compares the J2-2P/J2-3 ratios in inflammatory dermatoses and primary cutaneous T-cell lymphomas (PCTCLs). The Mann-Whitney U test showed that the J2-2P/J2-3 ratios were significantly higher in PCTCLs than in the control group (*p* = 0.00018, two-tailed). There were no significant differences between PCTCL subtypes (Figure 3B). Additionally, Pearson's linear and Spearman's rank-order correlations were performed to evaluate the relationship between the J2-2P/J2-3 ratios and the histological tumor cell percentages, but demonstrated no statistical correlation (*p* = 0.48) in Pearson's correlation; *p*= 0.98 in Spearman's correlation).

Based on the J2-2P/J2-3 ratios in PCTCL samples and the upper limit (0.72%) of controls, we divided the PCTCL samples into two groups: high (> 0.72%) and low (≤ 0.72%) J2-2P/J2-3 ratios. Twenty-five and eight PCTCL samples were in the high and low ratio groups, respectively, and their distinction needs further investigation.

### 3.4 Comparison between PCTCLs with high and low J2-2P/J2-3 ratios

In general, PCTCLs with a high J2-2P/J2-3 ratio had higher J2-2P (*p*-value = 0.0009) and J2-3 (*p*-value = 0.02) concentrations than PCTCLs with low J2-2P/J2-3 ratios. However, there was no statistically significant difference in PCTCL subtype (*p*-value = 0.07), age at diagnosis (*p*-value = 0.77), gender (*p*-value = 0.42), biopsy size (*p*-value = 1.00), tumor proportion in histology (*p*-value = 0.65), DNA quality (*p*-value = 0.62), and RNA concentrations (*p*-value = 0.24) between PCTCLs with high and low J2-2P/J2-3 ratios (Table 3). This indicates that the ratio is positively associated with the concentrations of the J2-2P and J2-3 sequences.

## 4. Discussion

Approximately 83% of primary cutaneous lymphomas were PCTCLs, according to a global meta-analysis 22. Worldwide, MF was the most common type of PCTCL (62%), despite variances by study and region. Regarding other types, SPTCL and PTCL were more prevalent in Asia 22. In our institute, MF constituted 82.6% of PCTCLs, and most MF cases were at the early stage 23. Moreover, pathological appearances may overlap features among certain subtypes of PCTCLs and benign mimics 24. Based on the low neoplastic T-cell infiltrates in early PCTCLs, a sensitive approach is crucial for early diagnosis and patient treatment.

Since T-cell malignancies are caused by a single T-cell clone, examining malignant T cells would reveal an identical TCR gene sequence, known as monoclonality 25. Consequently, molecular analyses for the monoclonality of TCR genes can support the diagnosis of PCTCLs, although monoclonality can sometimes develop in autoimmune diseases and reactive lesions 26-27. Among molecular tests, the standardized TCR primer sets from BIOMED-2/EuroClonality and multiplex PCR protocols have been widely employed to differentiate between PCTCLs and lymphoproliferative skin diseases 12,13,28. However, around 20% of PCTCLs failed to show monoclonality in the BIOMED-2 clonality assay 14,17. The sensitivities of the BIOMED-2 clonality assay for the early stages of PCTCLs, particularly early MF, could be lower than all PCTCL cases (TCRγ alone: 43-69.6%; TCRβ alone: 78.3-83%) 14,29. Although the BIOMED-2 multiplex PCR is a reliable and valuable diagnostic technique for patients with a preliminary suspicion of PCTCL, it is crucial to have alternative biomarkers or supplementary methods to facilitate accurate diagnosis or precise monitoring of PCTCLs.

While TCR exists as an alpha/beta or gamma/delta heterodimer, each T cell and its progeny possess a set of TCR genes uniquely rearranged due to the various combinations of V(D)J gene segments during T-cell maturation 25. On the contrary, non-recombined TCR sequences are characterized by the absence of a V (or D) gene segment, containing only a J gene segment joined to a constant (C) region segment. In contrast, the BIOMED-2 primers are designed to target the V (or D) and J gene segments, enabling the detection of various fully recombined TCR alleles. Consequently, the BIOMED-2 assay has predominantly bypassed these non-recombined TCR sequences 18. Interestingly, our recent study found high frequencies of non-recombined TCR sequences in the *TCRβ* repertoire sequencing, and the non-recombined *TCRβ* sequences, J2-2P, were most abundant in TCL samples 18. Herein, we previously examined concentrations of the J2-2P and J2-3 sequences via dPCR in blood or bone marrow samples of nine TCLs with either positive or negative results of BIOMED-2 TCR beta clonality. All nine cases had a high ratio of the J2-2P to J2-3 concentration, while non-TCL cases did not 18. Since the BIOMED-2 assay has fair detection rates for TCLs, we want to examine the novel biomarker (J2-2P/J2-3 ratio) for PCTCLs, a heterogeneous group with frequent diagnostic challenges.

Our current study demonstrated that the concentrations of J2-2P among all inflammatory dermatoses samples were consistently low (range: 0.035 to 0.07 copies/µL), and the ratios of the J2-2P to J2-3 concentrations averaged 0.21% (Table 2). The results of control samples indicated that the J2-2P sequences and the J2-2P/J2-3 ratios should be at low concentrations and ratios in benign or reactive cutaneous lesions. On the contrary, 25 PCTCL specimens exhibited a definitively high ratio of J2-2P/J2-3 (> 0.72%). When comparing PCTCLs with high and low J2-2P/J2-3 ratios, those with a high J2-2P/J2-3 ratio had statistically higher J2-2P and J2-3 concentrations. This suggests that the presence of the non-recombined J2-2P sequences in T cells was associated with the high expression. Although not statistically significant—likely due to the limited sample size—PCTCLs with a high J2-2P/J2-3 ratio exhibited a trend towards presenting in patients with MF and having higher RNA concentrations. These observations warrant further investigation in larger studies to confirm their clinical significance. Moreover, the biological or diagnostic meaning of the 8 PCTCL cases with low J2-2P/J2-3 ratios remains an area for continued studies. These low ratios may be explained by intra-tumoral heterogeneity, instances of extremely low neoplastic T-cell fractions that persist even after macro-dissection, variations in FFPE-derived RNA integrity, or distinct biological behaviors among certain PCTCL subtypes.

Since the non-recombined J2-2P and J2-3 sequences are derived from V(D)J recombination of *TCRβ* repertoire, the elevated concentrations of non-recombined sequences deserve further investigation into the potential mechanism and clinical impacts. Our previous report showed higher J2-2P/J2-3 ratios in blood or bone marrow from patients with TCLs and polyclonal *TCRβ* results 18. Our current study is the first report to investigate the existence of non-recombined TCR sequences in skin samples and their potential roles in PCTCLs with a negative BIOMED-2 result. The concentrations and ratios of J2-2P and J2-3 (non-recombined TCR sequences) might be possibly associated with disease status, therapeutic response, or prognosis, although we did not find such a relationship based on our limited clinical data and case number.

In addition, a highly sensitive method is crucial to quantitatively target J2-2P and J2-3 and supplement the moderate sensitivity of BIOMED-2 multiplex PCR for PCTCLs. Notably, dPCR performed well in detecting minimal residual disease for leukemia and lymphoma due to its high sensitivity to detect low amplicon numbers 30. In addition, dPCR enables the detection and absolute quantitation of rare targets with a higher sensitivity by partitioning samples into thousands or millions of individual PCR reactions, allowing for the amplification and detection of even a single target molecule 31. This absolute partitioning is highly advantageous for detecting the low absolute concentrations of J2-2P copies seen in certain clinical samples, mitigating the measurement reliability issues typically encountered in conventional qPCR. Herein, using the dPCR method to target the J2-2P and J2-3 sequences may be a good strategy to supplement the detection of PCTCLs after the BIOMED-2 clonality assay and provide an early diagnosis in small biopsy specimens and low tumor cellularity. With the improvement in detecting early cancer, patients with PCTCLs can largely benefit from personalized approaches and immunotherapy 32.

This study comes with several limitations. First, the study is a retrospective analysis of a limited-sized patient cohort and controls from a single medical facility, which may limit the generalizability of the results. Prospective, multi-center, large case cohorts, and large benign mimics are needed for further validation and clinical correlation. Second, we only selected clinicopathologically-proven PCTCL cases with a negative result of the BIOMED-2 clonality assay for testing, which did not inform the sensitivity of our method. Furthermore, while our control group of 11 inflammatory dermatoses provided a preliminary assessment of specificity, the exclusion of a larger cohort of non-PCTCL clinical mimics restricts our ability to fully define the diagnostic specificity of the J2-2P/J2-3 ratio. However, we aim to develop a supplementary method for the missing PCTCL cases in the BIOMED-2 clonality assay in this report. Third, we did not compare the current method to more advanced but expensive technologies, such as TCR gene high-throughput sequencing analysis. Although high-throughput sequencing might become more and more prevalent for clonality evaluations in PCTCLs 33-36, a recent report employing TCR sequencing in skin tissues demonstrated a sensitivity of 68% and a specificity of 100% in distinguishing PCTCLs from reactive conditions 35. These detection rates are not dominantly superior to those obtained with the BIOMED-2 clonality assay. Moreover, utilizing high-throughput sequencing for the TCR gene in evaluating clonality and T-cell fractions is much more time-consuming and expensive than the current dPCR method. Fourth, the limited number of non-MF PCTCL cases restricts our ability to draw definitive conclusions about the performance of this biomarker across all PCTCL subtypes, as explored in our subtype stratification.

## 5. Conclusion

In conclusion, our study shows that probing the non-recombined J2-2P and J2-3 sequences using dPCR is promising for adjunctively diagnosing PCTCLs with a negative BIOMED-2 result. The method can identify PCTCLs with a higher J2-2P/J2-3 ratio than those of reactive skin lesions and serves as supplementary testing for equivocal or negative results in the BIOMED-2 assay. The high concentrations of non-recombined TCR sequences may indicate clonal relatedness and warrant further investigation and exploration. In addition, prospective validation in large cohorts is critical before clinical metrics such as sensitivity, specificity, and predictive values can be fully assessed.

## Supplementary Material

Supplementary figures and tables.

## Figures and Tables

**Table 1:** The basic information and dPCR results of all PCTCLs.

No.	Gender	Diagnosis	TCRG (BIOMED-2)	TCRB (BIOMED-2)	Biopsy size (cm)	tumor cell (%)	DNA quality	RNA concentration (ng/µL)	J2-2P (HEX) Positives	J2-2P (HEX) DNA copies/µL	J2-3 (FAM) Positives	J2-3 (FAM) DNA copies/µL	J2-2P/J2-3(%)
1	M	PTCL	Polyclonal	-	0.7	40	400bp	157.9	0	0.035	173	12.13	0.29
2	F	PTCL	Polyclonal	-	0.4	20	200bp	17.2	9486	664.02	9735	681.45	97.44
3	M	PTCL	Polyclonal	Polyclonal	1.8	95	400bp	7.1	0	0.035	41	2.87	1.22
4	F	PTCL	Polyclonal	Polyclonal	1	85	400bp	141.9	7	0.48	780	54.58	0.88
5	M	SPTCL	Polyclonal	Polyclonal	3	10	400bp	775.6	3	0.21	2671	187.03	0.11
6	M	SPTCL	Equivocal	Polyclonal	2	10	400bp	9.5	3	0.23	839	68.07	0.36
7	F	SPTCL	Polyclonal	Polyclonal	1.5	8	400bp	12.8	0	0.035	100	6.99	0.50
8	F	SPTCL	Polyclonal	Polyclonal	4.3	40	400bp	1841.1	46	3.55	114	8.82	40.25
9	M	MF	Polyclonal	Polyclonal	0.8	6	400bp	42.4	2	0.14	42	2.94	4.76
10	M	MF	Polyclonal	Polyclonal	0.8	4	400bp	39	0	0.035	15	1.04	3.37
11	F	MF	Polyclonal	Polyclonal	0.8	16	300bp	22.9	0	0.035	0	0.035	NA*
12	M	MF	Polyclonal	Polyclonal	0.9	5.5	400bp	43.4	160	10.92	349	23.73	46.02
13	F	MF	Polyclonal	-	0.7	5	400bp	8.2	0	0.035	5347	374.3	0.01
14	F	MF	Polyclonal	-	1	10	400bp	19	2	0.14	23	1.66	8.43
15	M	MF	Polyclonal	Polyclonal	1	2	300bp	38.4	823	55.96	861	58.54	95.59
16	M	MF	Polyclonal	Polyclonal	0.9	5	400bp	101.6	338	22.98	399	27.13	84.70
17	M	MF	Polyclonal	-	1.2	3	300bp	15.5	0	0.035	870	59.16	0.06
18	M	MF	Polyclonal	Polyclonal	0.8	20	300bp	27.7	412	28.02	421	28.63	97.87
19	F	MF	Polyclonal	Polyclonal	0.5	15	400bp	29.6	18	1.34	1663	134.79	0.99
20	F	MF	Polyclonal	Polyclonal	0.7	12	400bp	36.7	128	14.82	349	23.73	62.45
21	M	MF	Polyclonal	Polyclonal	0.4	8	400bp	11.4	409	27.81	479	32.57	85.39
22	M	MF	Equivocal	Polyclonal	0.9	1	300bp	21.3	0	0.035	0	0.035	NA*
23	M	MF	Polyclonal	Polyclonal	0.5	3	400bp	12.8	350	23.8	488	33.18	71.73
24	M	MF	Polyclonal	-	0.8	2	400bp	8.5	5	0.35	669	46.84	0.75
25	F	MF	Polyclonal	Polyclonal	0.6	5	400bp	11.6	4	0.3	55	3.87	7.75
26	M	MF	Polyclonal	Polyclonal	0.5	6	300bp	12.5	0	0.035	162	11.36	0.31
27	F	MF	Polyclonal	Polyclonal	0.8	2	400bp	29.2	1	0.11	21	1.5	7.33
28	M	MF	Polyclonal	Polyclonal	1	4.5	400bp	28.6	0	0.035	60	4.2	0.83
29	F	MF	Polyclonal	Polyclonal	1	10	400bp	8.3	2	0.15	285	19.94	0.75
30	M	MF	Polyclonal	Polyclonal	0.7	5	400bp	10.5	2	0.14	53	3.69	3.79
31	M	MF	Polyclonal	-	0.7	20	400bp	85.4	282	20.81	297	21.93	94.89
32	F	MF	Polyclonal	-	0.5	10	300bp	59.8	6	0.44	59	4.31	10.21
33	F	MF	Polyclonal	Polyclonal	0.9	5	400bp	158.5	156	11.09	192	13.67	81.13
34	M	MF	Polyclonal	Polyclonal	0.5	30	400bp	68	3	0.27	548	50.21	0.54
35	F	MF	Polyclonal	-	0.7	10	400bp	123.3	5	0.36	229	16.6	2.17
Mean ± SD								115.35±327.70	361.51±1597.88	25.39±111.81	811.11±1,840.68	57.76±129.11	27.66±37.97

Abbreviations: M: male, F: female, MF: mycosis fungoides, PTCL: peripheral T-cell lymphoma, SD: standard deviation, SPTCL: subcutaneous panniculitis-like T-cell lymphoma, TCRG: TCRγ gene rearrangement, TCRB: TCRβ gene rearrangement, FAM: fluorescein, HEX: hexachloro-fluorescein, NA = not available. *the FAM-positive signals of J2-3 < 5

**Table 2:** The dPCR results for J2-2P and J2-3 of the control samples.

Control	Tissue	J2-2P (HEX) Positives	J2-3 (FAM) Positives	J2-2P (HEX) DNA copies/µL	J2-3 (FAM) DNA copies/µL	J2-2P/J2-3(%)
1	skin	0	2	0.035	0.14	NA*
2	skin	0	201	0.035	13.99	0.25
3	skin	0	87	0.035	6.38	0.55
4	skin	0	225	0.035	15.06	0.23
5	skin	1	1100	0.07	79.3	0.09
6	skin	0	1544	0.035	130.76	0.03
7	skin	0	1515	0.035	117.39	0.03
8	skin	0	303	0.035	21.25	0.16
9	skin	0	711	0.035	49.8	0.07
10	skin	0	142	0.035	9.71	0.36
11	skin	0	146	0.035	9.66	0.36
Mean				0.04	41.22	0.21
Standard deviation				0.01	46.97	0.17

*the FAM-positive signals of J2-3 < 5

**Table 3:** Comparison of PCTCL patient characteristics between high and low groups of the J2-2P/J2-3 ratios.

	PCTCL with negative BIOMED-2 (n = 33^a^)			
Characteristics	Total	High J2-2P/J2-3 ratio	Low J2-2P/J2-3 ratio	*P* value^b^
Case number	N = 33 (100)^c^	N = 25 (75.76)	N = 8 (24.24)	
Diagnosis				0.07
MF	25 (75.76)	21 (84.00)	4 (50.00)	
Non-MF	8 (24.24)	4 (15.00)	4 (50.00)	
Age at diagnosis , median (IQR)	61.00 (46.00-70.50)	61.00 (46.00-71.00)	63.50 (42.75-67.00)	0.77
Gender				0.42
Male	19 (57.58)	13 (52.00)	6 (75.00)	
Female	14 (42.42)	12 (48.00)	2 (25.00)	
Biopsy size				1.00
˃ 0.5cm	26 (78.79)	20 (80.00)	6 (75.00)	
≤ 0.5cm	7 (21.21)	5 (20.00)	2 (25.00)	
Tumor proportion				0.65
≥ 5%	26 (78.79)	19 (76.00)	7 (87.50)	
< 5%	7 (21.21)	6 (24.00)	1 (12.50)	
DNA quality				0.62
≥ 400 bp	27 (81.82)	21 (84.00)	6 (75.00)	
< 400 bp	6 (18.18)	4 (16.00)	2 (25.00)	
RNA concentration				0.24
≥ 20 ng/µL	19 (57.58)	16 (64.00)	3 (37.50)	
< 20 ng/µL	14 (42.42)	9 (36.00)	5 (62.50)	
J2-2P concentration				0.0009
≥ 0.3 copies/µL	17 (51.52)	17 (68.00)	0 (0.00)	
< 0.3 copies/µL	16 (48.48)	8 (32.00)	8 (100.00)	
J2-3 concentration				0.02
≥ 50 copies/µL	9 (27.27)	4 (16.00)	5 (62.50)	
< 50 copies/µL	24 (72.73)	21 (84.00)	3 (37.50)	

Abbreviations: MF, mycosis fungoides; IQR, interquartile range. ^a^We started with 35 PCTCL cases, but excluded 2 from analysis because their FAM-positive signals fell below the threshold of 5. ^b^Fisher's exact test was performed on all variables except the age at diagnosis, which was analyzed by the Mann-Whitney test. Fisher's exact test was utilized due to the small sample sizes across several categories. ^c^Data are expressed as the No. (%) of participants unless otherwise specified.

## Data Availability

The data presented in this study are provided within the manuscript or supplementary information files.
