# Supplementary material for: A digital polymerase chain reaction method targeting non-recombined T-cell receptor sequences aids in diagnosing primary cutaneous T-cell lymphomas
Source: J Cancer. 2026 Jul 13;17(7):1362–72. doi: 10.7150/jca.136694 (PMC13410567; doi:10.7150/jca.136694)
Supplement: Supplementary file 1 — Supplementary figures and tables. [file jcav17p1362s1.pdf]

Supplementary data

Supplementary Table S1. RT-PCR step1 conditions		
	Temperature	Time
Program 1	70℃	10min
Program 2	4℃	∞

Supplementary Table S2. RT-PCR step2 reagents	
Reagent	Volume (μL)
5 X first stand buffer	5.0
0.1 M DTT	5.0
Mg^{++} (25mM)	5.0
10 mM dNTP	2.5
Random hexamer (300ng/μL)	1.0
RNase Inhibitor	1.0
Reverse Transcriptase	1.0
RT-PCR step1 products	5.0
Total volume	25.5/tube

Supplementary Table S3. RT-PCR step2 conditions		
Program 1	25℃	10min
Program 2	42℃	60sec
Program 3	95℃	5min
Program 4	12℃	∞

Supplementary Table S4. dPCR reagents	
Reagent	Volume (μL)
H_2O	4.65
5X Master Mix (Roche)	3.0
20X JN SOL	0.75
F primer	2 (1+1)
R primer	2 (1+1)
Probe	1.6 (0.8+0.8)
cDNA	1
Total volume	15/tube

Supplementary Table S5. dPCR conditions			
Mode	Temperature	Time	
Program 1	95℃	5min	1 cycle
Program 2	95℃<br>58℃	50sec<br>90sec	50 cycles
Program 3	70℃	20min	1 cycle
Program 4	70℃	∞	

Supplementary Figures.

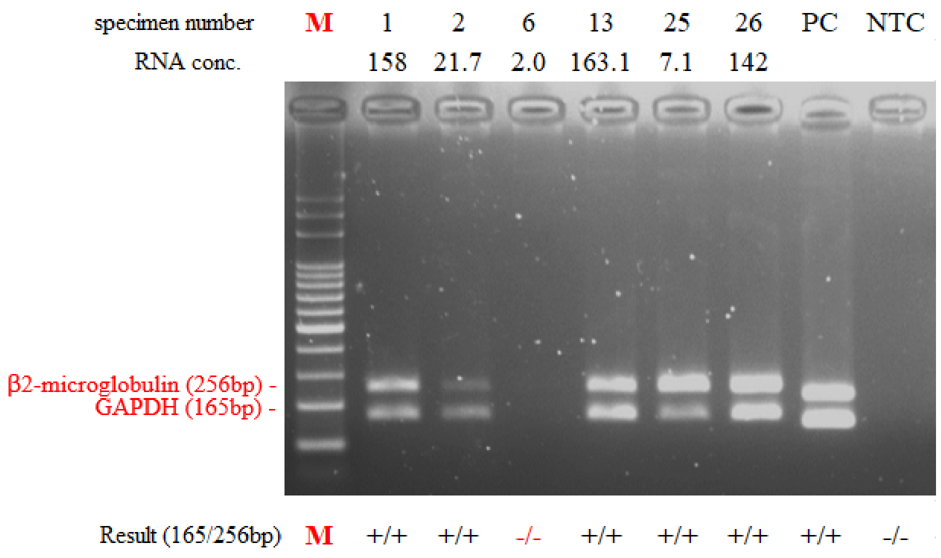

Supplementary Figure S1. The quality control of RT-PCR. GAPDH (165bp) and β2-microglobulin (256bp) were used as housekeeping genes to verify reverse transcription efficiency. If the two genes can be successfully amplified, reverse transcription is successful. If the internal control does not respond (like specimen number 6), further tissue slides must be re-cut to re-extract RNA and repeat the experiment. PC: Positive control (normal human cDNA); NTC: No template control.

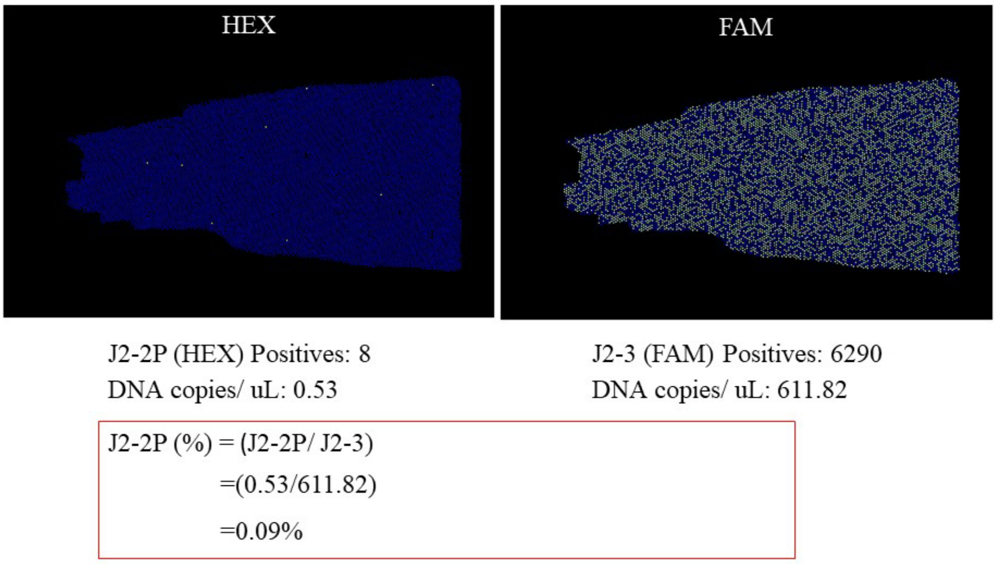

Supplementary Figure S2. An example of calculating the J2-2P/J2-3 ratio.

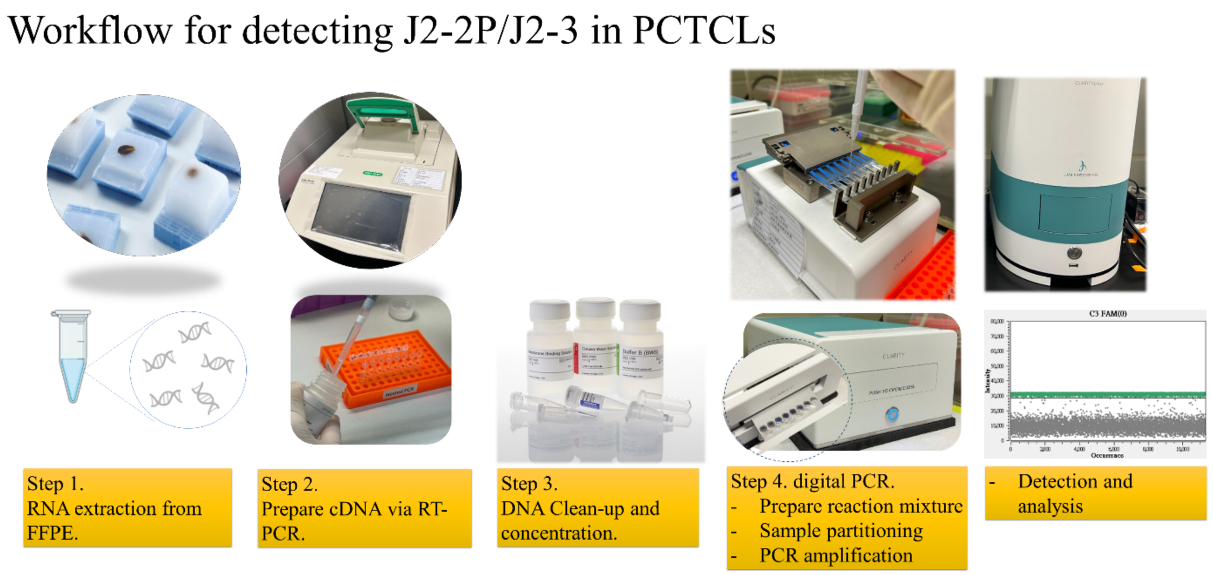

Supplementary Figure S3. The workflow from nucleic acid extraction to digital PCR (dPCR).

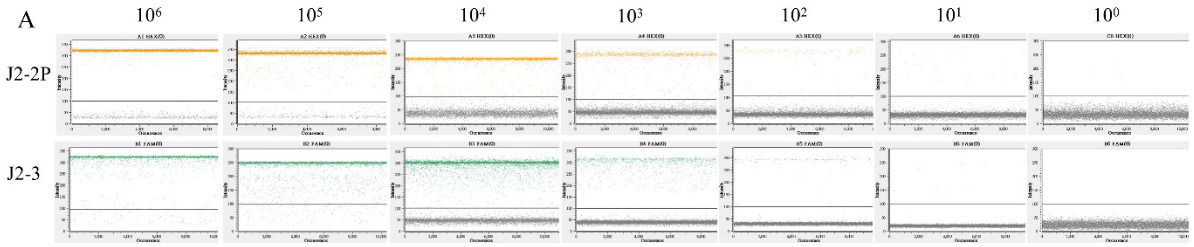

B

J2-2P/J2-3 Plasmid (copy number)	J2-2P (HEX) Positive	J2-2P DNA copies/μL	J2-3 (FAM) Positive	J2-3 DNA copies/μL
10^6	8650	1992.69	10798	3734.94
10^5	8299	2303.23	10852	3444.39
10^4	5396	528.29	5204	479.40
10^3	1217	96.75	1061	85.73
10^2	144	10.58	127	9.13
10^1	30	2.09	12	0.85
10^0	4	0.27	0#	0#

#Since zero values in correlation analysis and log transformations are unable to be defined, the zero values are not analyzed.

Supplementary Figure S4. Determination of limit of detection (LoD) for the J2-2P and J2-3 concentrations. (A) The 10-fold serial dilutions of the plasmid containing J2-2P and J2-3 (from 10^6 to 10^0 copies) were separately examined the concentrations of J2-2P and J2-3 via dPCR. Fluorescent signals (J2-2P probe: HEX, yellow color; J2-3: FAM, green color) were evenly distributed without background noises. (B) The optimal detection range of plasmid copy numbers for J2-2P and J2-3 was found to be 10^1 to 10^4 copies of plasmid, as signals were not detected at concentrations below 10^1 copies, and plateau effects were observed at concentrations over 10^4 copies.
